# Non-targeted metabolomics reveals myocardial metabolic alterations in epileptic rats

**DOI:** 10.1371/journal.pone.0346628

**Published:** 2026-05-21

**Authors:** Yue Hu, Yusen Wang, Qinghong Mao, Shisheng Zhu, Li Zhang

**Affiliations:** 1 Department of Basic Medical Sciences, Teaching and Research Office of Anatomy, Bijie Medical College, Bijie, China; 2 Department of Forensic Medicine, Chongqing Medical University, Chongqing, China; 3 Faculty of Basic Medical Sciences, Chongqing Medical and Pharmaceutical College, Chongqing, China; 4 School of Basic Medical Sciences, Chongqing University of Chinese Medicine, Chongqing, China; Sun Yat-Sen University, CHINA

## Abstract

Sudden unexpected death in epilepsy (SUDEP) is closely associated with cardiovascular dysfunction, yet its mechanisms remain unclear. Emerging evidence suggests that epilepsy-induced cardiac dysfunction may play a central role. Metabolomics provides a powerful approach to comprehensively characterize these metabolic alterations, enabling biomarker discovery and mechanistic insight into epilepsy-related cardiac injury. Here, we used UHPLC-OE-MS-based non-targeted metabolomics to systematically characterize myocardial metabolism in epileptic rats, aiming to identify epilepsy-related myocardial metabolic biomarkers, explore their biological significance, and elucidate mechanisms underlying epilepsy-induced cardiac dysfunction. In this study, we employed UHPLC-OE-MS-based non-targeted metabolomics to systematically profile myocardial metabolic changes in a pentylenetetrazol (PTZ)-induced epileptic rat model. Myocardial tissues from epileptic and control rats were analyzed using multivariate statistical methods, including principal component analysis (PCA) and partial least squares-discriminant analysis (PLS-DA), to identify significantly altered metabolites. KEGG pathway enrichment analysis was performed to elucidate the biological relevance of these metabolic disturbances. Histopathological examination revealed marked neuronal disorganization and myocardial injury in epileptic rats, characterized by cardiomyocyte swelling, nuclear pyknosis, and interstitial edema. Metabolomic analysis identified 127 differential metabolites, primarily involved in amino acid metabolism, glycerophospholipid metabolism, and energy metabolism. Notably, L-glutamine, L-phenylalanine, L-alanine, and β-alanine were significantly reduced, potentially contributing to glutamate/GABA imbalance, excitotoxicity, and oxidative stress. Additionally, dysregulated glycerophospholipids (e.g., LysoPC, PC, and PE) and altered pantothenate and CoA biosynthesis indicated compromised membrane integrity and impaired energy metabolism in epileptic myocardium. ROC analysis demonstrated excellent diagnostic performance of key metabolites (AUC > 0.98), highlighting their potential as biomarkers for epilepsy-related cardiac dysfunction.

## Background

Epilepsy is a prevalent neurological disorder characterized by recurrent episodes of abnormal neuronal discharges, leading to disrupted neuronal activity and various neurological symptoms [1]. According to the World Health Organization (WHO), epilepsy is one of the most significant neurological disorders affecting global health, with approximately 50 million people currently diagnosed worldwide and an estimated 5 million new cases reported annually. The condition is associated with a relatively high mortality rate, with a global average of 1.74 deaths per 100,000 individuals—2.09 per 100,000 in males and 1.64 per 100,000 in females [2].

Sudden unexpected death in epilepsy (SUDEP), one of the most severe complications of epilepsy, represents the leading cause of mortality in epilepsy patients [3–5]. SUDEP is defined as a sudden, unexpected, non-traumatic, and non-drowning death in individuals with epilepsy, in which postmortem examination fails to reveal a structural or toxicological cause. Globally, the incidence of SUDEP is estimated at approximately 1 case per 1,000 epilepsy patients annually [6], with a higher prevalence in China, reaching approximately 2.03 cases per 1,000 person-years [7]. The precise mechanisms underlying SUDEP remain incompletely elucidated, with one of the primary challenges being the lack of an ideal experimental model that accurately replicates human pathophysiological characteristics. Although animal models have proven valuable for investigating SUDEP mechanisms and exploring potential interventions, species-specific physiological and pathological differences limit their ability to fully recapitulate the complex pathological processes seen in human cases.

Studies indicate that SUDEP involves multiple physiological mechanisms, including cardiac dysfunction, respiratory abnormalities, autonomic nervous system dysregulation, and neuronal damage [8]. Central respiratory depression due to brainstem dysfunction is considered a key trigger for SUDEP [9]. When epileptic seizures affect the brainstem, they can impair respiratory center function, potentially leading to fatal respiratory arrest or respiratory failure. In recent years, myocardial injury has been increasingly recognized as a critical factor contributing to SUDEP. Seizure-induced physiological stress can significantly impact cardiac function, particularly through excessive activation of the autonomic nervous system and cardiovascular stress responses. This may result in arrhythmias, metabolic disturbances, and myocardial structural damage, thereby elevating the risk of sudden cardiac death [10,11]. Pathological studies have identified structural abnormalities, such as myocardial hypertrophy and fibrosis, in some SUDEP cases, suggesting a strong correlation between epilepsy-induced myocardial injury and SUDEP [12].

Therefore, investigating epilepsy-related myocardial metabolic alterations and identifying biomarkers of myocardial injury are essential for assessing SUDEP risk. Early detection of myocardial injury-associated metabolites may provide valuable insights into cardiovascular risk assessment in epilepsy patients and facilitate the development of targeted clinical interventions, ultimately reducing SUDEP incidence and improving patient outcomes.

Ultra-high-performance liquid chromatography–high-resolution mass spectrometry (UHPLC-OE-MS) has emerged as a powerful tool in biomarker discovery. By integrating high-resolution chromatographic separation with highly sensitive mass spectrometric detection, this technique enables rapid and efficient analysis of complex biological samples, including metabolites and proteins, with high throughput, sensitivity, and accuracy [13]. UHPLC-OE-MS facilitates untargeted metabolomics analysis, allowing for a comprehensive screening of metabolic profiles and the identification of previously unrecognized disease-related biomarkers. Compared to traditional targeted analytical methods, untargeted metabolomics provides a more holistic view of disease pathophysiology, supporting early diagnosis and risk assessment [14]. Currently, metabolomics technology has become an important tool for the study of metabolic abnormalities in complex diseases, and has been widely used in the screening of biomarkers for neurological diseases and cardiovascular diseases [15].

**Fig 1:**
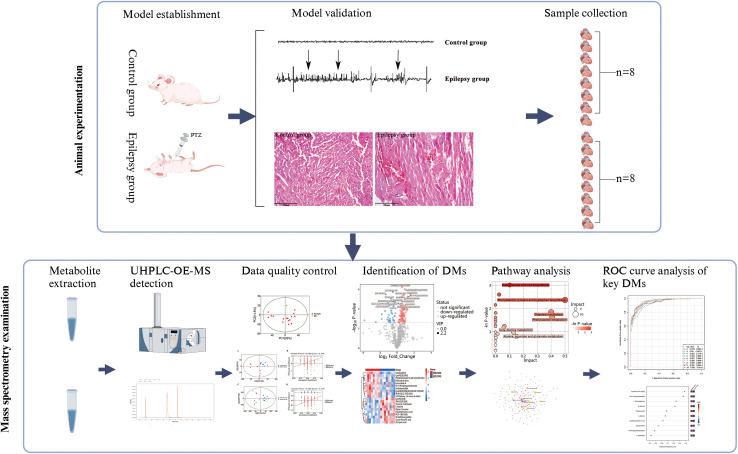
Schematic illustration of study.

In this study, UHPLC-OE-MS technology was employed to comprehensively characterize the metabolic profiles of myocardial tissue in an epileptic rat model. Differential metabolites between the epilepsy and control groups were identified, and the potential mechanisms underlying epilepsy-induced myocardial injury and metabolic disturbances were investigated. As illustrated in the graphical abstract (Fig 1), the workflow encompassed metabolite profiling, differential analysis, and pathway enrichment to delineate the metabolic perturbations associated with epilepsy. The findings of this study provide novel candidate metabolic biomarkers and theoretical insights that may facilitate the early diagnosis and intervention of epilepsy-related cardiac dysfunction.

## Materials and methods

### Animals and ethical statement

This study utilized male Sprague-Dawley (SD) rats of specific pathogen-free (SPF) grade, weighing 220 ± 50 g, provided by the Animal Center of Chongqing Medical University. The rats were housed in a controlled environment with a temperature of 22°C, humidity of 60%, and a 12-hour light/dark cycle, with free access to food and water. After an acclimation period, the experiments commenced. The animals were randomly assigned to two groups: the control group (N = 15) and the epilepsy group (N = 15). All experimental procedures were conducted in strict accordance with the Guidelines for the Care and Use of Laboratory Animals and were approved by the Institutional Animal Care and Use Committee (IACUC) of Bijie Medical College. Humane endpoints were applied during the entire study. Animals were monitored daily for general health and seizure severity. Euthanasia was performed if animals exhibited (i) >20% body weight loss, (ii) inability to access food or water, (iii) moribund appearance with lack of righting reflex. Once criteria were met, rats were euthanized immediately (within 1 hour) via intraperitoneal injection of sodium pentobarbital (150 mg/kg). No animals reached the predefined humane endpoint criteria during PTZ kindling. Therefore, no rats required euthanasia for humane reasons during model establishment.

### Establishment of the epilepsy model

Epilepsy was induced in the experimental group according to the method described by Zhang et al [16]. Briefly, rats received intraperitoneal (i.p.) injections of a 1% pentylenetetrazol (PTZ) solution at a dose of 35 mg/kg, administered every other day for a total of 11 injections. The control group received an equal volume of normal saline at the same time points. Seizure severity was evaluated based on Racine’s seizure scoring system: Stage 0: No abnormal response; Stage I: Facial muscle twitching or vibrissae tremors; Stage II: Rhythmic nodding or wet dog shakes; Stage III: Unilateral limb clonus; Stage IV: Bilateral forelimb clonus with rearing; Stage V: Loss of balance, falling, rolling, or jumping, accompanied by generalized tonic-clonic seizures and possible mortality. Successful model establishment was confirmed after completion of the PTZ kindling protocol, corresponding to an average of approximately 21 days from the first PTZ injection to confirmation of the epilepsy model.

### Electroencephalography (EEG) recording

EEG recordings were performed using the RM6240C multichannel physiological signal acquisition and processing system (Chengdu Instrument Factory, Chengdu, China). Rats were placed in the EEG recording apparatus immediately after PTZ or saline injection, and continuous EEG monitoring was conducted for 30 minutes. The primary EEG parameters analyzed included seizure-like discharge frequency, amplitude, and duration. Successful model establishment was confirmed by the presence of sustained epileptiform discharges, which corresponded to PTZ-induced behavioral seizure manifestations such as myoclonic jerks, nodding, focal clonus, or generalized tonic-clonic seizures. No characteristic discharges or abnormal behaviors were observed in the control group.

### Tissue collection

Rats were anesthetized by intraperitoneal injection of sodium pentobarbital (2%, 65 mg/kg). After deep anesthesia was confirmed, the rats were placed in the supine position and secured on the operating table. The thoracic cavity was opened via a median thoracotomy using sterile scissors and forceps to expose the heart. Cardiac blood was then collected directly from the heart using a sterile syringe. Subsequently, 50 mL of sterile ice-cold normal saline was slowly infused through the left ventricle using a sterile 100 mL syringe for transcardial perfusion. When the limbs and visceral organs became pale, indicating adequate perfusion, and no respiration or heartbeat was observed, myocardial and brain tissues were rapidly harvested for subsequent analyses. Portions of the tissues were immediately snap-frozen in liquid nitrogen and stored at −80°C for metabolomic analysis, while the remaining tissues were fixed in 10% neutral-buffered formalin for 24–48 h for histological examination.

### Histological analysis

Fixed myocardial and brain tissues were dehydrated through a graded ethanol series, cleared in xylene, embedded in paraffin, and sectioned. The sections were deparaffinized in xylene and rehydrated through a descending ethanol gradient. Hematoxylin staining was performed for 5 minutes, followed by washing with tap water to develop the blue coloration. The sections were then differentiated in 0.5% acid alcohol, blued in an alkaline solution, and counterstained with eosin for 2 minutes. After dehydration and clearing, the sections were mounted. Stained sections were observed under a light microscope, and images were captured for analysis of tissue structure and pathological changes.

### UHPLC-OE-MS analysis

#### Sample preparation.

A 25 mg portion of myocardial tissue was weighed and homogenized in 500 μL extraction solution (methanol: acetonitrile: water = 2:2:1, v/v) containing an isotope-labeled internal standard mixture. The homogenization was performed at 35 Hz for 4 minutes, followed by ultrasonication for 5 minutes in an ice-water bath. This process was repeated twice. The samples were then incubated at −40°C for 1 hour, followed by centrifugation at 12,000 rpm (13,800 × g, 8.6 cm rotor radius) for 15 minutes at 4°C. The supernatant was collected for UHPLC-OE-MS analysis, and quality control (QC) samples were prepared by pooling equal aliquots of all supernatants.

#### Instrumental analysis.

Metabolomic profiling was conducted using a Vanquish ultra-high-performance liquid chromatography (UHPLC) system (Thermo Fisher Scientific) equipped with a Waters ACQUITY UPLC BEH Amide column (2.1 mm × 100 mm, 1.7μm). The mobile phase consisted of: Solvent A: Aqueous phase containing 25 mmol/L ammonium acetate and 25 mmol/L ammonium hydroxide; Solvent B: Acetonitrile.

The autosampler was maintained at 4°C, and the injection volume was 2 μL. Mass spectrometric detection was performed using an Orbitrap Exploris 120 mass spectrometer (Thermo Fisher Scientific) controlled by Xcalibur software (version 4.4). Data acquisition included both full-scan MS and data-dependent MS/MS with the following parameters: Sheath gas flow rate: 50 Arb, Aux gas flow rate: 15 Arb, Capillary temperature: 320°C, Full MS resolution: 60000, MS/MS resolution: 15000,

Collision energy: 10/30/60 in normalized collision energy (NCE) mode, Spray voltage: 3.8 kV (positive ion mode) or −3.4 kV (negative ion mode).

#### Data processing.

Raw data files were converted into mzXML format using ProteoWizard software and subsequently processed using an in-house R package based on the XCMS algorithm for peak detection, extraction, alignment, and integration. Metabolite annotation was performed by matching spectra against the BiotreeDB (V2.1) in-house secondary MS database, with a scoring cutoff of 0.3.

#### Identification of differential metabolites.

All annotated metabolites were classified into defined confidence levels, including:(A) identification confirmed by authentic standards or NMR; (Bi–Biii) high-confidence MS/MS-based identification using experimental or in silico spectral databases; (Ci–Ciii) MSⁿ-based identification with varying degrees of spectral matching; and (D) MS-based annotation relying on accurate mass information. Processed data were imported into SIMCA 14.1 software for principal component analysis (PCA) to assess data quality and instrumental stability based on QC sample distribution. Partial least squares-discriminant analysis (PLS-DA) and orthogonal PLS-DA (OPLS-DA) were subsequently conducted. Differential metabolites were identified based on OPLS-DA variable importance for the projection (VIP) scores, combined with an independent-samples Student’s t-test (p < 0.05). The metabolomics data generated in this study are provided in S1 Table.

#### Metabolic pathway analysis.

Metabolic pathway analysis was performed using the online platform MetaboAnalyst(https://www.metaboanalyst.ca/MetaboAnalyst/ModuleView.xhtml), which integrates pathway enrichment and topology analyses to identify key metabolic pathways associated with the differential metabolites.

### Statistical analysis

All statistical analyses were performed using SPSS 21.0 software. Data were expressed as mean ± standard deviation (SD). Group comparisons were conducted using an independent-sample t-test, with p < 0.05 considered statistically significant. Metabolites with VIP scores >1 in OPLS-DA analysis were regarded as potential differential metabolites.

## Results

### EEG detection results

**Fig 2:**
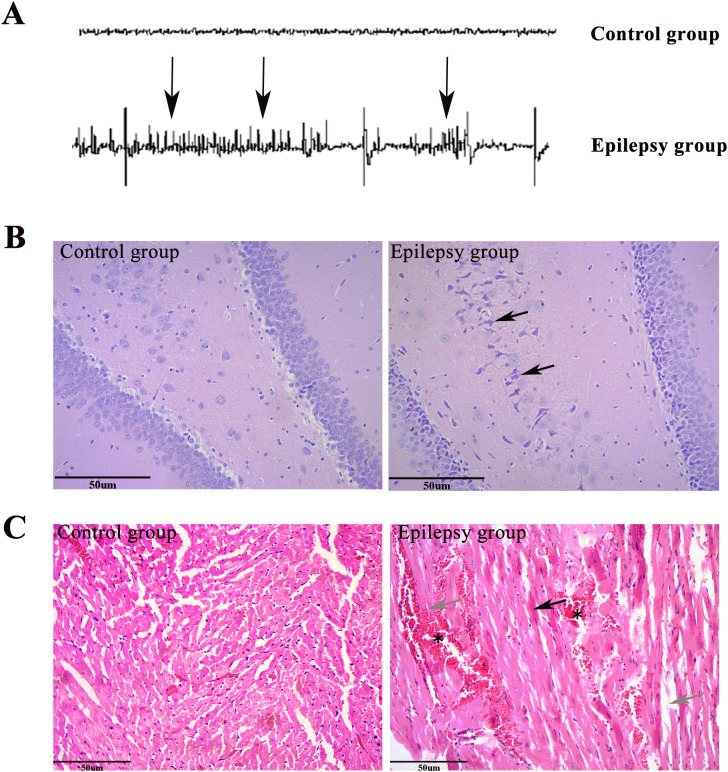
Electroencephalographic and histopathological characterization of the control and epilepsy groups. **(A)** Representative EEG traces recorded from the control and epilepsy groups. Compared with the control group, the epilepsy group exhibited abnormal epileptiform discharges, indicated by black arrows, reflecting enhanced pathological electrical activity. **(B)** Representative hematoxylin and eosin (HE)-stained sections of brain tissue from the control and epilepsy groups (magnification, × 200; scale bar = 50 µm), showing the histopathological changes associated with epilepsy. **(C)** Representative HE-stained sections of myocardial tissue from the control and epilepsy groups (magnification, × 200; scale bar = 50 µm), illustrating the myocardial histopathological alterations observed following epilepsy.

EEG recordings in the epilepsy group revealed significant abnormal electrical activity (Fig 2A). The results indicated a high frequency of epileptiform discharges, primarily characterized by intermittent spike waves and sharp waves, as indicated by black arrows. In contrast, EEG recordings in the control group exhibited normal background electrical activity without epileptiform discharges. These findings confirm the successful establishment of the epilepsy model.

### Morphological examination results

Hematoxylin and eosin (HE) staining revealed significant pathological changes in the brain and myocardial tissues of epileptic rats (Fig 2B and 2C). Representative pathological alterations are indicated by arrows/markers to facilitate visual identification. In the epilepsy group, brain tissue showed disorganized neuronal arrangement, and some neurons exhibited shrunken cell bodies and nuclear pyknosis (black arrows), accompanied by tissue loosening and edema. In contrast, the control group showed normal brain tissue architecture, with regularly arranged neurons and intact cellular morphology, without obvious pathological abnormalities.

Similarly, myocardial tissue in the epilepsy group showed mild cardiomyocyte swelling, cytoplasmic condensation (black arrows), cardiomyocyte fragmentation (gray arrows), as well as interstitial edema and hemorrhage (*). In contrast, the control group exhibited a normal myocardial structure, with neatly arranged cardiomyocytes and no obvious pathological alterations. The arrows/markers added in Fig 2C specifically highlight these representative myocardial lesions, thereby improving the clarity and interpretability of the histopathological findings. These results suggest that epilepsy induces pathological injury in myocardial tissue.

### UHPLC-OE-MS-based metabolomic profiling of myocardial tissue

#### Data quality control.

**Fig 3:**
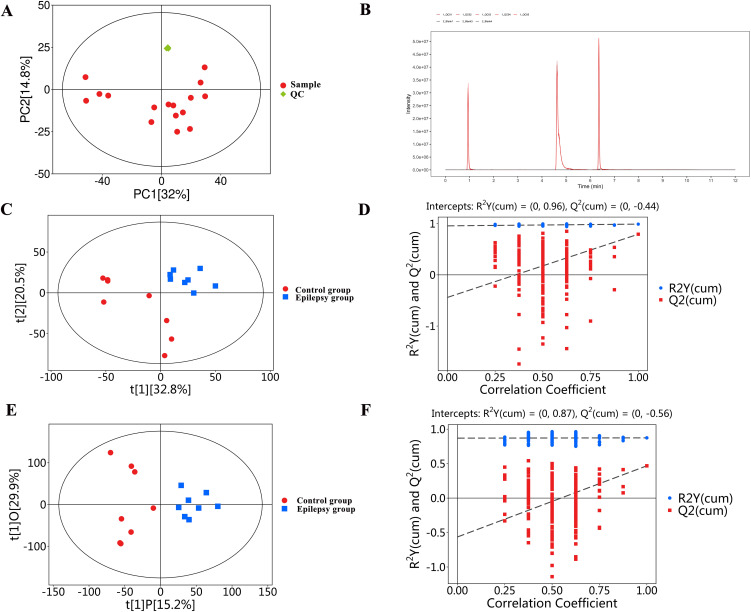
Data quality control and metabolic profiling of myocardial tissue. (A) Principal component analysis (PCA) score plot of quality control (QC) samples and study samples. The tight clustering of QC samples indicates high analytical reproducibility, good instrument stability, and reliable overall data quality. (B) Representative overlap chromatograms of QC samples, demonstrating good retention time consistency and signal stability during the analytical run. (C) Partial least squares-discriminant analysis (PLS-DA) score plot showing a clear separation between the epilepsy and control groups, indicating distinct metabolic profiles in myocardial tissue. (D) Permutation test (200 permutations) used to validate the PLS-DA model. The intercepts of R² (0.96) and Q² (−0.44) support the robustness of the model and indicate that the model was not overfitted. (E, F) Orthogonal partial least squares-discriminant analysis (OPLS-DA) score plot and corresponding model validation results further demonstrating a significant metabolic distinction between the epilepsy and control groups, with strong model interpretability and reliability.

In theory, QC samples should exhibit minimal variation. However, minor errors may arise during sample extraction and instrumental analysis. To monitor analytical reproducibility and system stability, pooled QC samples were repeatedly injected throughout the analytical sequence. Principal component analysis (PCA) demonstrated that the QC samples were tightly clustered (Fig 3A), with no obvious outliers, indicating good instrument stability and high reliability of the metabolomics dataset. In addition, the overlap chromatograms of QC samples showed highly consistent peak patterns and retention times (Fig 3B), further supporting the stability and reproducibility of the analytical platform.

#### Screening of differential metabolites in myocardial tissue.

PLS-DA analysis of normalized data showed that the epilepsy and control groups were distributed in distinct metabolic clusters (Fig 3C), suggesting significant differences in metabolite profiles between the two groups. To evaluate potential overfitting, a 7-fold cross-validation strategy combined with a 200-time permutation test was applied. The permutation test yielded R² and Q² intercepts of 0.96 and −0.44, respectively (Fig 3D), indicating that the PLS-DA model was robust and not overfitted. OPLS-DA analysis was further performed to maximize group discrimination while removing orthogonal (non-class-related) variation. The OPLS-DA model exhibited good explanatory power and predictive performance, further confirming the presence of significant metabolic differences between the epilepsy and control groups (Fig 3E and Fig 3F). Importantly, PLS-DA and OPLS-DA were primarily used for pattern recognition and visualization, rather than as the sole criteria for metabolite selection.

By analyzing the VIP scores of the OPLS-DA model, a total of 127 potential differential metabolites were identified, among which 86 metabolites were up-regulated and 41 metabolites were down-regulated in the epilepsy group (S2 Table).

**Fig 4:**
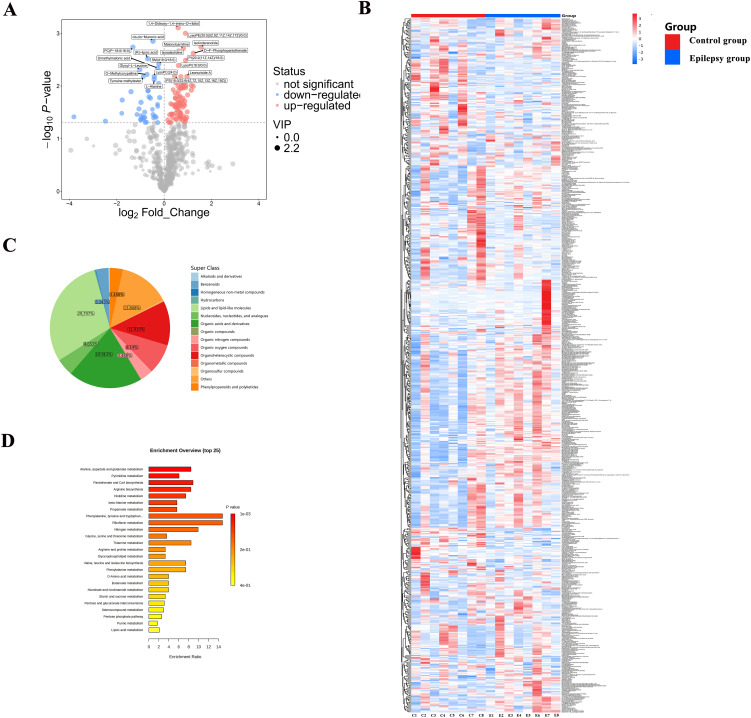
Screening and classification of differential metabolites in myocardial tissue. (A) Volcano plot of differential metabolites detected between the epilepsy and control groups, showing a total of 127 significantly altered metabolites, including 86 upregulated and 41 downregulated metabolites in the epilepsy group. (B) Hierarchical clustering heatmap of differential metabolites, demonstrating clear segregation between the epilepsy and control samples based on their metabolic signatures. (C) Chemical classification of the differential metabolites into 15 categories, with lipids and lipid-like molecules (29.797%) and organic acids and derivatives (19.913%) representing the two most abundant classes. (D) Pathway enrichment analysis of the top 25 significantly enriched metabolic pathways associated with the differential metabolites; the x-axis indicates the enrichment ratio, and the y-axis lists the corresponding pathway names.

These results are visualized in the volcano plot (Fig 4A). Hierarchical clustering analysis of differential metabolites (Fig 4B) clearly separated the epilepsy and control groups, highlighting consistent and group-specific metabolic signatures. The classification of differential metabolites (Fig 4C) revealed 15 categories, with lipids and lipid-like molecules (29.797%) and organic acids and derivatives (19.913%) being the most enriched. Furthermore, pathway enrichment analysis of the top 25 significant pathways is presented in Fig 4D, where enrichment ratios are shown on the x-axis and pathway names on the y-axis, providing insights into the metabolic perturbations associated with epilepsy.

#### Metabolic pathway analysis of differential metabolites in myocardial tissue of epileptic rats.

**Fig 5:**
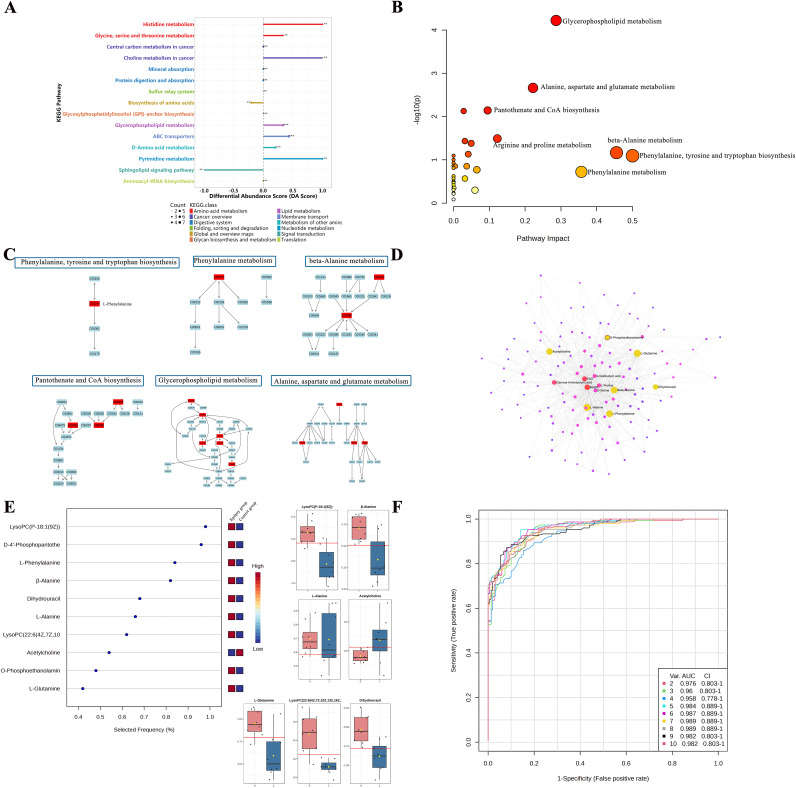
Metabolic pathway analysis and biomarker evaluation of differential metabolites. **(A)** Differential pathway abundance score analysis showing the overall variation in pathway activity between the epilepsy and control groups. **(B)** Bubble plot of significantly altered metabolic pathways, highlighting the most relevant pathways associated with myocardial metabolic disturbances in epilepsy. **(C)** Representative compound–relation maps of significant metabolites, illustrating the relationships between key metabolites and their associated biochemical pathways. **(D)** Metabolite–metabolite interaction network showing the interconnections among differential metabolites and their potential coordinated roles in metabolic regulation. **(E)** Feature importance ranking of metabolites, identifying the most stable and discriminative metabolites contributing to group separation. **(F)** Receiver operating characteristic (ROC) curve analysis evaluating the diagnostic performance of key metabolites and confirming their potential value as candidate biomarkers.

To further elucidate the biological significance of differential metabolites, the 127 identified metabolites were mapped to the KEGG database for pathway enrichment analysis. A total of 81 metabolic pathways were involved (S3 Table). Differential abundance score analysis (Fig 5A) revealed significant downregulation of the sphingolipid metabolism pathway, whereas amino acid metabolism (including histidine metabolism, glycine-serine-threonine metabolism, and amino acid biosynthesis), glycerophospholipid metabolism, and ABC transporter pathways were significantly upregulated.

Key metabolic pathways with an impact score >0.1 (Fig 5B) included glycerophospholipid metabolism, alanine–aspartate–glutamate metabolism, pantothenate and CoA biosynthesis, arginine and proline metabolism, β-alanine metabolism, phenylalanine–tyrosine–tryptophan biosynthesis, and phenylalanine metabolism. Representative compound–relation maps and metabolic conversion pathways are illustrated in Fig 5C, while metabolite–metabolite interaction networks are presented in Fig 5D.

Further metabolite feature importance ranking analysis (Fig 5E) identified acetylcholine, L-glutamine, L-phenylalanine, O-phosphoethanolamine, dihydrouracil, L-alanine, β-alanine, LysoPC (22:6(4Z,7Z,10Z,13Z,16Z,19Z)), D-4’-phosphopantothenate, and LysoPC (P-18:1(9Z)) as the most stable and discriminative key metabolites, with markedly different expression levels between the epilepsy and control groups. These findings suggest that epilepsy-induced myocardial injury is closely associated with disturbances in membrane lipid metabolism, energy metabolism, and amino acid metabolism.

Finally, ROC curve analysis (Fig 5F) confirmed the diagnostic potential of these metabolites. AUC values reached 0.984 (95% CI: 0.889–1.0) with the top five metabolites, and exceeded 0.989 (95% CI: 0.889–1.0) when eight or more variables were included, indicating that the selected metabolite panel possesses high discriminatory and predictive power for epilepsy-related cardiac dysfunction.

## Discussion

Epilepsy, a prevalent neurological disorder, not only affects the central nervous system but also exerts significant effects significant effects on multiple organ systems, including the cardiovascular system. SUDEP is one of the leading causes of mortality among epilepsy patients. Research suggests that SUDEP may be closely associated with the impact of epileptic seizures on cardiac function, particularly in patients with persistent or severe epilepsy, where cardiac arrest or heart failure is a major cause of death [17]. In this study, untargeted metabolomics was employed to systematically analyze the metabolic characteristics of myocardial tissue in epileptic rats. Multiple significantly altered metabolites and related pathways were identified, providing new insights into the relationship between epilepsy and myocardial metabolic dysregulation.

Our results indicate that epilepsy significantly alters the metabolic state of myocardial tissue, particularly in amino acid metabolism, lipid metabolism, and energy metabolism. We detected significant changes in the levels of metabolites such as acetylcholine, L-glutamine, L-phenylalanine, O-phosphoethanolamine, dihydrouracil, L-alanine, β-alanine, LysoPC, D-4’-phosphopantothenate, and LysoPC in the myocardial tissue of epileptic rats, suggesting that epilepsy may induce myocardial dysfunction by disrupting metabolic homeostasis. KEGG pathway enrichment analysis identified 127 differential metabolites involved in 81 metabolic pathways, with significant changes in glycerophospholipid metabolism, alanine–aspartate–glutamate metabolism, pantothenate and CoA biosynthesis, arginine and proline metabolism, β-alanine metabolism, phenylalanine–tyrosine–tryptophan biosynthesis, and phenylalanine metabolism. These metabolic disruptions may play a critical role in epilepsy-induced myocardial injury.

Specifically, the levels of L-glutamine, L-phenylalanine, L-alanine, and β-alanine were markedly decreased in the myocardium of epileptic rats. Amino acid metabolism is essential for energy production, neurotransmitter synthesis, and cellular repair, and its disruption may contribute to glutamate/GABA imbalance, excitotoxicity, and neuroinflammation [18]. As a carnosine precursor, β-alanine supports antioxidant capacity, pH buffering, and mitochondrial energy, potentially mitigating oxidative stress and metabolic imbalance; however, excessive β-alanine may deplete taurine, impairing calcium homeostasis and membrane stability, thereby increasing myocardial vulnerability [19]. L-glutamine is critical for myocardial energy metabolism and antioxidant defense, and its reduction is linked to impaired cardiac function and oxidative stress [20]. Phenylalanine plays a central role in myocardial metabolism; its decrease in epilepsy may worsen metabolic imbalance and neurotransmitter dysfunction, whereas higher baseline phenylalanine in heart failure patients predicts better CRT response, reflecting preserved amino acid and energy metabolism [21]. Additionally, L-arginine, a nitric oxide precursor, protects the myocardium by enhancing endothelial function, energy metabolism, and antioxidant defense, reducing oxidative stress and improving cardiac resilience in myocardial infarction, epilepsy, and heart failure models [22]. Collectively, these findings suggest that disruptions in amino acid metabolism, particularly involving L-glutamine, L-phenylalanine, L-alanine, and β-alanine, synergistically contribute to epilepsy-induced myocardial injury and highlight these metabolites, along with L-arginine, as potential therapeutic targets for cardiac protection.

Glycerophospholipid metabolism was significantly altered in the myocardial tissue of epileptic rats, with notable upregulation of lipid molecules such as lysophosphatidylcholine (LysoPC), phosphatidylethanolamine (PE), phosphatidylcholine (PC), and phosphatidylserine (PS), while acetylcholine and O-PEA levels were significantly reduced. Glycerophospholipids are crucial components of cell membranes, and their dysregulation may result in membrane instability, impaired signal transduction, and myocardial dysfunction [23]. LysoPC, a critical signaling molecule associated with atherosclerosis, acute inflammation, and chronic inflammation, may drive inflammatory responses and provoke arrhythmias upon upregulation in epileptic myocardial tissue. [24]. Meng et al. [25] identified significant enrichment of glycerophospholipid metabolism in a rat myocardial infarction model using metabolomics, demonstrating accumulation of LysoPC in the serum of MI rats, along with a marked reduction in their upstream metabolite PC. Studies have shown that O-PEA can interfere with mitochondrial respiration and energy metabolism, ultimately affecting ATP production and cellular homeostasis [26]. Additionally, in ischemia-reperfusion injury, an increase in O-PEA has been associated with cellular damage and membrane dysfunction, while its regulation may help restore membrane integrity and mitigate ischemic damage [27]. Current evidence suggests that O-PEA may contribute to epilepsy-induced myocardial injury by influencing metabolic, phosphorylation, and oxidative stress pathways. However, further research is needed to elucidate its precise mechanisms. The role of glycerophospholipid metabolism in myocardial injury is complex and multi-faceted, as its dysregulation affects membrane stability, signal transduction, and cell survival processes [28].

In this study, we observed significant disruptions in the biosynthetic pathway of pantothenic acid and coenzyme A (CoA) in the myocardial tissue of epileptic rats. Both pantothenic acid and CoA are essential for myocardial energy metabolism, fatty acid oxidation, oxidative stress response, and membrane stability. Research has shown that CoA deficiency is a critical pathological factor contributing to cardiac dysfunction [29]. Epilepsy may exacerbate myocardial injury by impairing CoA synthesis, leading to metabolic disturbances and worsening cardiac damage. Therefore, targeting pantothenic acid and CoA metabolism presents a promising therapeutic approach for epilepsy-related myocardial injury, potentially enhancing cardiac function in epilepsy patients and reducing the risk of epilepsy-associated cardiac mortality.

Significant alterations were observed in several key metabolites involved in energy metabolism in the myocardium of epileptic rats, particularly those related to cofactor availability and redox-related metabolic regulation. For instance, metabolites such as nicotinamide mononucleotide (NMN), NADP, FAD, lipoic acid, and pantothenate/CoA-related intermediates showed significant changes, indicating disturbances in metabolic regulation and enzymatic cofactor homeostasis. Although these molecules are not direct energy substrates, they play crucial roles in maintaining mitochondrial enzyme activity and metabolic flexibility. Their coordinated alterations suggest that, despite the absence of significant changes in direct energy substrates like fatty acids, acylcarnitines, glucose, or lactate, the regulation of energy metabolism in the heart was still significantly affected, likely due to the disruption in cofactor availability, which in turn impacts myocardial energy homeostasis.

Furthermore, the changes in L-glutamine and β-alanine, although relatively minor, suggest their involvement in redox metabolism, contributing to antioxidant responses and cellular repair. Although the antioxidant capacity of these amino acids is weaker compared to cysteine and glutathione, their reduction may be closely related to oxidative stress and metabolic imbalance in the myocardium.

### Limitations of the study

Despite the valuable insights provided by our analysis, we acknowledge several limitations in the current study. First, the absence of direct functional cardiac assessments represents an important limitation. Future studies should incorporate electrocardiographic and echocardiographic evaluations to provide a more comprehensive assessment of cardiac function and to better correlate metabolic alterations with functional myocardial impairment. Second, this study relied exclusively on untargeted metabolomic profiling as an exploratory approach, and no targeted quantitative metabolomics or independent validation cohort was included to verify the observed changes in key metabolites. Therefore, the identified metabolites should be interpreted as preliminary candidate biomarkers and mechanistic clues, and further confirmation in independent cohorts and by targeted metabolomic approaches will be necessary to improve the robustness, reproducibility, and translational relevance of these findings. Third, although we discussed the potential biological significance of the altered metabolites and pathways, the current study still lacks direct experimental evidence to fully delineate the precise upstream and downstream mechanisms by which these metabolic disturbances affect cardiomyocyte stability and function. In particular, the mechanistic links between reduced metabolic fluxes, mitochondrial function, redox balance, membrane integrity, and myocardial cellular injury require further in-depth investigation. Finally, validation in alternative epilepsy models is also needed to determine the generalizability of our findings across different experimental settings. Addressing these issues in future studies will further strengthen the interpretation, rigor, and applicability of the present work.

## Conclusion

In conclusion, epilepsy induced significant myocardial injury in rats, characterized by structural abnormalities and interstitial edema. Untargeted metabolomics revealed marked alterations in amino acid metabolism, glycerophospholipid metabolism, and energy metabolism, with notable decreases in L-glutamine, L-phenylalanine, L-alanine, and β-alanine. These findings suggest that metabolic remodeling may be involved in epilepsy-associated myocardial injury. Alterations in LysoPC and PC further indicate possible disturbances in membrane lipid homeostasis and myocardial function. However, the specific mechanistic roles of these metabolites, as well as their potential clinical or therapeutic relevance, require further validation through functional experiments and clinical studies.

## Supporting information

S1 Table Raw metabolomics data. (XLSX)

S2 Table Differential metabolites between epilepsy group and control group. (XLS)

S3 Table Differential metabolite pathways between epilepsy group and control group. (XLSX)
